# Dr. Ransom Walker

**Published:** 1894-10

**Authors:** Edwin T. Darby


### Obituary.

### DR. RANSOM WALKER.

Died at Owego, N. Y., July 31, 1894, of apoplexy, Ransom Walker, D.D.S., in the seventy-sixth year of his age.

Dr. Walker was born in Greene, Chenango County, N. Y., October 23, 1818. He received his early education in the public schools of his native town and at Cortland Academy at Homer, N. Y.

As he approached early manhood he felt impelled to enter the Christian ministry, and his subsequent education was along these lines, but later he abandoned that idea, and entered upon the study of dentistry, being a pupil of the late Amos Wescott, of Syracuse. During the time of his pupilage the New York College of Dental Surgery was established at Syracuse, at which institution he attended one course of lectures, and the following year removed to Owego, where he began the practice of dentistry. He very soon acquired a large and lucrative practice.

During the winter of 1865–66 he left his practice to attend a course of lectures at the Pennsylvania College of Dental Surgery, from which he graduated in the spring of 1866. He then returned to Owego and resumed his professional work, which he pursued almost uninterruptedly until he was stricken, while at his operating-chair, August 20, 1892. This was followed by another attack, a few days later, which so disabled him that he was compelled to abandon his professional labors. Although he somewhat rallied from these attacks and was able to keep about, he never regained the entire use of his hands. The final stroke came but a few days before his death.

Dr. Walker was a dentist of more than ordinary ability, possessing as he did manipulative skill in an eminent degree.

During the first twenty years of his practice he received into his office as pupils many young men, and he was regarded as a faithful and conscientious preceptor. Among those who received their early training at his hands were O. E. Hill, of Brooklyn; Edwin T. Darby, of Philadelphia; Charles H. Darby, of St. Joseph, Mo.; and Frank B. Darby, of Elmira, N. Y. Dr. Walker was among the first to improve the character of dental amalgams, and after years of experimentation he put upon the market, in 1866, an alloy composed of gold, silver, platinum, and tin, which was known as Walker's excelsior amalgam. When a good man dies it is but fitting that we make mention of his worth, and Dr. Walker was a good man. He inherited from his ancestry a good constitution and a good name, but not riches; but by industry, honesty, and prudence he attained a competency.

The sense of justice was never more marked in any man than in him. His moral and religious character was above reproach. He was a true friend, a loving husband, a public-spirited citizen, a Christian gentleman. As I pen these memorial lines of my departed preceptor and friend I pause to hang upon his tombstone a garland rich in appreciation of kindness received at his hands.

Dr. Walker was married to Mary Snider, of Virgil, N. Y., September 3, 1845. She still survives him.

EDWIN T. DARBY.

